# A comparison of cisplatin cumulative dose and cisplatin schedule in patients treated with concurrent chemo-radiotherapy in nasopharyngeal carcinoma^☆^

**DOI:** 10.1016/j.bjorl.2019.04.008

**Published:** 2019-05-22

**Authors:** Mete Gundog, Hatice Basaran, Oktay Bozkurt, Celalettin Eroglu

**Affiliations:** a Erciyes University, Department of Radiation Oncology, Kayseri, Turkey; b Erciyes University, Department of Medical Oncology, Kayseri, Turkey

**Keywords:** Cumulative cisplatin dose, Chemotherapy schedule, Nasopharyngeal carcinoma, Dose cumulativa de cisplatina, Esquema de quimioterapia, Carcinoma nasofaríngeo

## Abstract

**Introduction:**

Three-weekly cisplatin dose is accepted for standard treatment for concurrent chemo-radiotherapy in nasopharyngeal carcinoma. However, different chemotherapy schedules are presented in the literature.

**Objective:**

We intend to compare toxicity and outcomes of high dose 3-weekly cisplatin versus low dose weekly-cisplatin and cumulative dose of cisplatin in the patients with nasopharyngeal carcinoma.

**Methods:**

98 patients were included in the study, between 2010 and 2018. Cumulative doses of cisplatin (≥200 mg/m^2^ and <200 mg/m^2^) and different chemotherapy schedules (weekly and 3-weekly) were compared in terms of toxicity and survival. Besides prognostic factors including age, gender, T category, N category and radiotherapy technique were evaluated in uni-multivariate analysis.

**Results:**

Median follow-up time 41.5 months (range: 2–93 months). Five year overall survival, local relapse-free survival, regional recurrence-free survival and distant metastasis-free survival rates were; 68.9% vs. 90.3% (*p* = 0.11); 66.2% vs. 81.6% (*p* = 0.15); 87.3% vs. 95.7% (*p* = 0.18); 80.1% vs. 76.1% (*p* = 0.74) for the group treated weekly and 3 weekly, respectively. There was no statistically significant difference between groups. Five year overall survival, local relapse-free survival, regional recurrence-free survival and distant metastasis-free survival rates were; 78.2% vs. 49.2% (*p* = 0.003); 75.8% vs. 47.9% (*p* = 0.055); 91% vs. 87.1% (*p* = 0.46); 80% vs. 72.2% (*p* = 0.46) for the group treated ≥200 mg/m^2^ and <200 mg/m^2^ cumulative dose cisplatin. There was statistically significant difference between groups for overall survival and there was close to being statistically significant difference between groups for local relapse-free survival. Age, gender, T category, N category, chemotherapy schedules were not associated with prognosis in the uni-variety analysis. Radiotherapy technique and cumulative dose of cisplatin was associated with prognosis in uni-variate analysis (HR = 0.21; 95% CI: 0.071–0.628; *p* = 0.005 and HR = 0.29; 95% CI: 0.125–0.686; *p* = 0.003, respectively). Only cumulative dose of cisplatin was found as an independent prognostic factor in multivariate analysis (HR = 0.36; 95% CI: 0.146–0.912; *p* = 0.03). When toxicities were evaluated, such as hematological toxicity, dermatitis, mucositis, nausea and vomiting, there were no statistically significant differences between cumulative dose of cisplatin groups (<200 mg/m^2^ and ≥200 mg/m^2^) and chemotherapy schedules (3-weekly and weekly). But malnutrition was statistically significant higher in patients treated with 3-weekly cisplatin compared with patients treated with weekly cisplatin (*p* = 0.001).

**Conclusion:**

A cisplatin dose with ≥200 mg/m^2^ is an independent prognostic factor for overall survival. Chemotherapy schedules weekly and 3-weekly have similar outcomes and adverse effects. If patients achieve ≥200 mg/m^2^ dose of cumulative cisplatin, weekly chemotherapy schedules may be used safely and effectively in nasopharyngeal carcinoma patients.

## Resumo

**Introdução:**

Três doses semanais de cisplatina com quimiorradioterapia concomitante são aceitas como o tratamento padrão para carcinoma nasofaríngeo. No entanto, diferentes esquemas quimioterápicos são recomendados na literatura científica.

**Objetivo:**

Comparar a toxicidade e os resultados de 3 doses altas semanais de cisplatina versus dose baixa semanal de cisplatina em pacientes com carcinoma nasofaríngeo e verificar a dose cumulativa de cisplatina.

**Método:**

Foram incluídos 98 pacientes, entre 2010 e 2018. As doses cumulativas de cisplatina (≥200 mg/m^2^ e <200 mg/m^2^) e diferentes esquemas de quimioterapia (semanal e a cada 3 semanas) foram comparadas em termos de toxicidade e sobrevida. Além disso, fatores prognósticos incluindo idade, sexo, categoria T, categoria N e técnica de radioterapia foram avaliados na análise uni-multivariada.

**Resultados:**

O tempo médio de seguimento foi de 41,5 meses (intervalo: 2–93 meses). Sobrevida global de cinco anos, sobrevida livre de recidiva local, sobrevida livre de recidiva regional e sobrevida livre de metástases a distância foram: 68,9% vs. 90,3% (*p* = 0,11); 66,2% vs. 81,6% (*p* = 0,15); 87,3% vs. 95,7% (*p* = 0,18); e 80,1% vs. 76,1% (*p* = 0,74) para os grupos tratados semanalmente e 3 x/semana, respectivamente. Não houve diferença estatisticamente significante entre os grupos. Taxas de sobrevida global, sobrevida livre de recidiva local, sobrevida livre de recidiva regional e sobrevida livre de metástases à distância em cinco anos foram; 78,2% vs. 49,2% (*p* = 0,003); 75,8% vs. 47,9% (*p* = 0,055); 91% vs. 87,1% (*p* = 0,46); 80% vs. 72,2% (*p* = 0,46) para o grupo tratado com ≥200 mg/m^2^ e <200 mg/m^2^ de dose cumulativa de cisplatina. Houve diferença estatisticamente significante entre os grupos para sobrevida global e houve uma diferença quase estatisticamente significante entre os grupos para sobrevida livre de recidiva local. Idade, sexo, categoria T, categoria N e esquemas de quimioterapia não foram associados ao prognóstico na análise univariada. A técnica de radioterapia e dose cumulativa de cisplatina foram associadas ao prognóstico na análise univariada (HR = 0,21; IC 95%: 0,071 ± 0,628; *p* = 0,005 e HR = 0,29; IC 95%: 0,125 ± 0,686; *p* = 0,003, respectivamente). Apenas a dose cumulativa de cisplatina foi considerada um fator prognóstico independente na análise multivariada (HR = 0,36; IC 95%: 0,146 ± 0,912; *p* = 0,03). Quando as toxicidades foram avaliadas, como toxicidade hematológica, dermatite, mucosite, náusea e vômito, não houve diferença estatisticamente significante entre a dose cumulativa dos grupos cisplatina (<200 mg/m^2^ e ≥200 mg/m^2^) e esquemas de quimioterapia (semanal e a cada 3 semanas). Entretanto, a desnutrição foi estatisticamente maior em pacientes tratados com cisplatina a cada 3 semanas em comparação com pacientes tratados com cisplatina semanalmente (*p* = 0,001).

**Conclusão:**

Uma dose de cisplatina ≥200 mg/m^2^ é fator prognóstico independente para sobrevida global. Os esquemas de quimioterapia semanais e a cada 3 semanas têm resultados e efeitos adversos semelhantes. Se os pacientes atingirem uma dose cumulativa ≥200 mg/m^2^ de cisplatina, os esquemas semanais de quimioterapia podem ser usados com segurança e eficácia em pacientes com carcinoma nasofaríngeo.

## Introduction

Nasopharyngeal carcinoma (NPC) is a malignancy of the head and neck endemic to south China, Southeast Asia and the Mediterranean region. Radiotherapy (RT) is the basic treatment for early-stage NPC, because of the high radio-sensitivity.^1^ However, locally advanced NPC is related with greater risk of loco-regional recurrence and distant metastasis.^1^, ^2^ Concurrent chemo-radiotherapy has been established as the standard of care treatment of locally advanced NPC on the basis of several studies demonstrating improvement in overall survival (OS),^3^, ^4^, ^5^, ^6^, ^7^, ^8^, ^9^ progression-free survival (PFS),^6^, ^7^, ^10^ disease-free survival (DFS),^8^ loco-regional^10^ and distant control compared to RT alone. For locally advanced NPC, combined chemotherapy with RT may prolong overall survival with a 5 year absolute survival benefit of 4%.^3^, ^4^, ^11^

National Comprehensive Cancer Network (NCCN) guidelines recommend platinum-based concurrent chemo-radiotherapy (CCRT) as the first line treatment for NPCs, because systemic therapies are more effective in NPC than other solid cancers.^12^, ^13^ The standard protocol in North America is the Intergroup-0099 regimen consisting of cisplatin 100 mg/m^2^ administered on days 1, 22 and 43, concurrently with RT, and followed by adjuvant cisplatin and 5-fluorourasil.^1^, ^6^ The NPC-0099 clinical trial demonstrated that high cisplatin dosage is more effective than RT alone for advanced NPC patients in terms of OS and PFS, but this regimen is associated with significant acute toxicity that can limit dose delivery.^14^ Adjuvant chemotherapy has frequently been omitted due to cumulative toxicity, and until recently, studies have not demonstrated clear clinical benefit supporting its use.^15^

In the literature, trials have shown seriously acute adverse reactions in patients with locally advanced laryngeal cancer that utilized high dose cisplatin both in post-operative chemo-radiation and organ-protective definitive chemo-radiotherapy.^15^, ^16^, ^17^, ^18^ In addition, updated in 2013, radiation therapy oncology group (RTOG) 91–11 trial demonstrated a 10 year cumulative rate of Grade 3–5 late toxicity of 33% and high incidence of death unrelated to larynx cancer in patients treated with cisplatin based chemo-radiotherapy for larynx preservation.^19^ Despite toxicity of 3-weekly high dose cisplatin protocol, low-dose weekly cisplatin protocols have been thought to result in less toxicity in different trials.^10^ A phase III randomized trial revealed that a weekly regimen (cisplatin 40 mg/m^2^) concurrent with radiotherapy was easier to tolerate for patients and showed survival benefits compared to RT alone.^10^

Concurrent chemo-radiotherapy is the standard therapy for locally advanced NPC, however optimal schedule for cisplatin dose has not been established. In this retrospective population-based study, we aim to compare toxicity and outcomes of 3-weekly high dose cisplatin versus low-dose weekly cisplatin in patients with NPC.

## Methods

### Patient selection

The diagnosis of NPC was confirmed by a multidisciplinary tumor board before initiating the treatment. Ninety eight patients with NPC treated with concurrent chemo-radiotherapy between years 2010 and 2018 were included. Inclusion criteria included histologically confirmed NPC by biopsy, no distant metastasis, normal hepatic functions (serum total bilirubin ≤1.6 mg/dL and serum transaminase levels <2.5 times higher than upper limit); normal renal functions (serum creatinine ≤1.5 mg/dL and creatinine clearance ≥60 mL/min); normal complete blood count level (haemoglobin >10 g/dL, white blood cells ≥4000 μL, platelet ≥100,000 μL); Karnofsky Performance Status (KPS) score of >70; and receiving only cisplatin during concurrent chemo-radiotherapy. This study was approved by local research ethics committee and written informed consent was provided by all patients (Local Research Ethics Committee ID: 2019/160 and date: 06.03.2019).

### Clinical staging

Staging was determined by clinical examination of head and neck, nasoendoscopy with direct fiber optic, Magnetic Resonance Imaging (MRI) and Positron Emission Computed Tomography (PET-CT). All patients were restaged according to the 8th edition of the American Joint Committee on Cancer (AJCC) system.^20^

### Treatment

2/3D conformal radiotherapy technique was performed before 2012 and IMRT technique was performed after 2012. Standard dose and fractionation for 2/3D conformal RT was 70 Gy to the primary tumor; 50–60 Gy to involved nodes in 35 daily fractions. The total dose and fraction for IMRT were 70 Gy/2.12 Gy per fraction for high risk planning target volume (primary tumor volume and involved nodes), 60 Gy/1.8 Gy per fraction for intermediate risk planning target volume and 54 Gy/1.65 Gy per fraction for low risk planning target volume. Cisplatin regimen consists of intravenous infusion of 100 mg/m^2^ every 3 weeks or 50 mg/m^2^ intravenous infusion weekly. Chemotherapy was started at the first radiation treatment day for all patients.

Toxicity information was recorded by medical staff during treatment and collected from patients’ charts. Acute toxicity evaluation of the treatment was performed according to National Cancer Institute Common Toxicity Criteria version 3.0.^21^

### Follow up

Patient follow up was defined from the first day of treatment to the last examination or death. Both MRI and PET-CT were performed to evaluate the response to treatment on the 3rd month after the last treatment day. The evaluation of the response to treatment was done according to Response Evaluation Criteria in Solid Tumors (RECIST criteria).^22^ The response to treatment was classified as Complete Response (CR), Partial Response (PR), Stable Disease (SD) or Progressive Disease (PD). If the response was complete, the patients were monitored every 3 months for the first 2 years with MRI and their follow-up examinations were performed every 6 months thereafter until death.

### Statistical analysis

The site of the first clinical relapse was accepted as local failure, if the relapse site is in the nasopharynx. The site of first clinical relapse was accepted as regional failure, if the relapse site is in the nodal region. If the relapse site was beyond the above-mentioned areas, we accepted it as distant failure. The duration of Disease Free Survival (DFS), Local Recurrence-Free Survival and Regional Recurrence-Free Survival (LRFS and RRFS) and distant metastasis-free survival (DMFS) were calculated from day 1 of the treatment until treatment failure is documented. Overall survival (OS) was calculated from day 1 of the treatment until death or the date of the last follow up. The statistical analysis of the data was performed by using IBM SPSS Statistics 22.0 (IBM Corp., Armonk, New York, USA). All data were expressed as means ± SD unless otherwise stated and controlled for normality using Shapiro–Wilk test. Chi-square and Fisher's exact tests were used to compare categorical variables such as different age, gender, T stage, N stage, and chemotherapy regimen. The survival time between groups was compared with Kaplan–Meier analysis and log-rank tests. Differences were considered significant at *p* < 0.05. The effective factors on overall survival and loco-regional relapse-free survival were analyzed using multivariate Cox regression (Backward/Wald method) model. Removal probability for stepwise was taken as *p* < 0.10. The value of *p* < 0.05 denoted statistical significance.

## Results

### Patient characteristics

From 2010 to 2018, a total of 98 (28 female and 70 male) eligible patients were evaluated. The median age was 49.5 years (range 16–75). Of the 98 patients, 17 (17.3%), 28 (28.6%), 20 (20.4%) and 33 (33.7%) had T_1_, T_2_, T_3_, and T_4_ stage cancers, respectively. Of the 98 patients, 20 (20.4%), 28 (28.6%), 38 (38.8%) and 12 (12.2%) had N_0_, N_1_, N_2_, and N_3_ stage cancers, respectively. Of the 98 patients, 19 (19.4%), 36 (36.7%) and 43 (43.9%) had Stage II, Stage III and Stage IV_A_ cancers, respectively. Sixty-one patients (62.2%) received weekly-cisplatin and 37 (37.8%) patients received 3-weekly cisplatin. Five of 98 patients (5.1%) were treated with 2/3D conformal RT technique. 93 of 98 patients (94.9%) were treated with IMRT technique. After chemo-radiotherapy 80 patients (81.6%) were evaluated as complete response and 18 patients (18.4%) were evaluated as partial response. A cumulative dose of ≥200 mg/m^2^ was achieved in 46 of 61 (74%) patients in weekly cisplatin group and all the patients (100%) in 3 weekly cisplatin group received a cumulative dose of ≥200 mg/m^2^. Median treatment day was found to be 48 days (range: 30–87 days). The characteristics of patients are summarized in Table 1.

**Table 1:** Comparison of patient's characteristics between groups.

		Weekly group *n* = 61 (%)	3-Weekly group *n* = 37 (%)	Total *n* = 98 (%)	*p*
Gender	Female	16 (26.2)	12 (32.4)	28 (28.6)	0.64^a^
	Male	45 (73.8)	25 (67.6)	70 (71.4)	
Age	≤50	28 (45.9)	27 (73)	55 (56.1)	0.01^a^
	>50	33 (54.1)	10 (27)	43 (43.9)	
T category	T1	13 (21.3)	4 (10.8)	17 (17.3)	0.16^b^
	T2	20 (32.8)	8 (21.6)	28 (28.6)	
	T3	12 (19.7)	8 (21.6)	20 (20.4)	
	T4	16 (26.2)	17 (45.9)	33 (33.7)	
N category	N0	14 (23)	6 (16.2)	20 (20.4)	0.30^b^
	N1	16 (26.2)	12 (32.4)	28 (27.5)	
	N2	26 (42.6)	12 (32.4)	38 (38.8)	
	N3	5 (8.2)	7 (18.9)	12 (12.2)	
RT technique	2/3D-RT	4 (6.6)	1 (2.7)	5 (5.1)	0.64^a^
	IMRT	57 (93.4)	36 (97.3)	93 (94.9)	
TNM stage	II	16 (26.2)	3 (8.1)	19 (19.6)	0.01^b^
	III	25 (41.0)	11 (29.7)	36 (36.7)	
	IVA	20 (32.8)	23 (62.2)	43 (43.9)	
Cumulative dose	≥200 mg/m^2^	45 (73.8)	37 (100)	82 (83.7)	<0.001^a^
	<200 mg/m^2^	16 (26.2)	0 (0)	16 (16.3)	

2/3D-RT, two or 3-dimensional radiotherapy; IMRT, intensity modulated radiotherapy; TNM, T and N categories according to the 8th edition of American Joint Commission on Cancer staging system.

a Fisher's exact test.

b Chi-square.

### Toxicity

Grade 1–2 hematological toxicity was found in 88 of all patients (89.8%) and Grade 3–4 hematological toxicity was found in 10 of all patients (10.2%). Six of the patients with Grade 3–4 hematological toxicity were in the 3 weekly cisplatin group and 4 of the patients with Grade 3–4 toxicity were in the weekly cisplatin group. There was no statistically significant difference between the two groups (*p* = 0.17). Grade 1–2 mucositis was found in 57 of all patients (57.2%) and Grade 3–4 mucositis was found in 41 of all patients (41.8%). Thirteen of the patients with Grade 3–4 mucositis were in 3-weekly cisplatin group and 28 of the patients with Grade 3–4 mucositis were in the weekly cisplatin group. There was no statistically significant difference between in the two groups (*p* = 0.39). Eighty seven of all patients (88.8%) experienced Grade 1–2 dermatitis and 11 of all patients (11.2%) experienced Grade 3–4 dermatitis. Nine patients with Grade 3–4 dermatitis were in the weekly cisplatin group and 2 patients with Grade 3–4 dermatitis were in the 3-weekly cisplatin group. There was no statistically significant difference between the two groups (*p* = 0.199). The most frequently determined toxicity was malnutrition. Compared to weekly-regimen, the 3-weekly cisplatin regimens induced significantly more severe nutrient toxicity. Sixty nine of all patients (69/98) experienced malnutrition. The average percentage of malnutrition in the weekly cisplatin group was 59% (36/61) and in the 3 weekly cisplatin group 89.2% (33/37). The malnutrition was not determined in 25 patients (41%) in the weekly cisplatin group and was not determined in only 4 patients (10.8%) in the 3-weekly cisplatin group. There was statistically significant difference between groups in terms of malnutrition (*p* = 0.001). The weekly cisplatin group was experienced less nutrient toxicity. The characteristics of patients’ toxicity are summarized in Table 2.

**Table 2:** Comparison of toxicity between the weekly and 3-weekly group.

		Weekly group *n* = 61 (%)	3-Weekly group *n* = 37 (%)	Total *n* = 98 (%)	*p*^a^
Hematological toxicity	Grade 1–2	57 (93.4)	31 (83.8)	88 (89.8)	0.17
	Grade 3–4	4 (6.6)	6 (16.2)	10 (10.2)	
Mucositis	Grade 1–2	33 (54.1)	24 (64.9)	57 (58.2)	0.39
	Grade 3–4	28 (45.9)	13 (35.1)	41 (41.8)	
Dermatitis	Grade 1–2	52 (85.2)	35 (94.6)	87 (88.8)	0.19
	Grade 3–4	9 (14.8)	2 (5.4)	11 (11.2)	
Nausea and vomiting	Grade 1–2	31 (50.8)	24 (64.9)	48 (56.1)	0.21
	Grade 3–4	30 (49.2)	13 (35.1)	50 (43.9)	
Malnutrition	Grade 1–2	25 (41)	4 (10.8)	29 (29.6)	0.001
	Grade 3–4	36 (59)	33 (89.2)	69 (70.4)	

a Fisher's exact test value.

Cumulative high-dose cisplatin did not show statistically significant difference in Grade 3–4 toxicities including hematological toxicity, mucositis, dermatitis, and malnutrition compared to cumulative low-dose cisplatin (*p* = 1.00; *p* = 0.09; *p* = 0.38; *p* = 1.00 respectively). For nausea and vomiting, the difference between the groups was close to being statistically significant (*p* = 0.051). The comparison between the toxicities and delivered cumulative dose cisplatin is summarized in Table 3.

**Table 3:** Comparison of the delivered cumulative cisplatin dose and toxicity.

		Patients with cisplatin ≥200 mg/m^2^ *n* = 82 (%)	Patients with cisplatin <200 mg/m^2^ *n* = 16 (%)	Total *n* = 98 (%)	*p*^a^
Hematological toxicity	Grade 1–2	73 (89)	15 (93.8)	88 (89.8)	1.00
	Grade 3–4	9 (11)	1 (6.3)	10 (10.2)	
Mucositis	Grade 1–2	51 (62.2)	6 (37.5)	57 (58.2)	0.096
	Grade 3–4	31 (37.8)	10 (62.5)	41 (41.8)	
Dermatitis	Grade 1–2	74 (90.2)	13 (81.3)	87 (88.8)	0.381
	Grade 3–4	8 (9.8)	3 (18.7)	11 (11.2)	
Nausea and vomiting	Grade 1–2	50 (61)	5 (31.3)	55 (56.1)	0.05
	Grade 3–4	32 (39)	11 (68.7)	43 (43.9)	
Malnutrition	Grade 1–2	24 (29.3)	5 (31.3)	29 (29.6)	1.00
	Grade 3–4	58 (70.7)	11 (68.7)	69 (70.4)	

a Fisher's exact test value.

### Treatment outcomes

Median follow up was found to be 41.5 months (range: 2–93 months). Local recurrence was observed in 23 of 98 patients. The local recurrence rate was 19.4% (19/61) in the weekly cisplatin group and 4.1% (4/37) in the 3-weekly cisplatin groups (*p* = 0.02) (Table 4). The regional-recurrence was observed in 8 of 98 patients. The regional-recurrence rate was 7.1% (7/61) in the weekly-cisplatin group and 1% (1/37) in the 3-weekly-cisplatin groups (*p* = 0.25) (Table 4). Distant metastasis was observed in 17 of 98 patients. The distant metastasis rate was 11.2% (11/61) in the weekly cisplatin group and 6.1% (6/37) in the 3-weekly cisplatin groups (*p* = 1.00) (Table 4).

**Table 4:** Comparison of the treatment outcomes between groups.

	Cisplatin ≥200 mg/m^2^	Cisplatin <200 mg/m^2^	Total		Cisplatin Weekly	Cisplatin 3 weekly	Total	
	*n* (%)	*n* (%)	*n* (%)	*p*	*n* (%)	*n* (%)	*n* (%)	*p*
*Treatment response*								0.42
Partial	14 (17.1)	4 (25)	18 (18.4)	0.48	13 (21.3)	5 (13.5)	18 (18.4)	
Complete	68 (82.9)	12 (75)	80 (81.6)		48 (78.7)	32 (86.5)	80 (81.6)	
*Local recurrence*								0.02
−	66 (80.5)	9 (56.3)	75 (76.5)	0.03	42 (68.9)	33 (89.2)	75 (76.5)	
+	16 (19.5)	7 (43.7)	23 (23.5)		19 (31.1)	4 (10.8)	23 (23.5)	
*Regional recurrence*								0.25
−	76 (92.7)	14 (87.5)	90 (91.9)	0.61	54 (88.5)	36 (97.3)	90 (91.8)	
+	6 (7.3)	2 (12.5)	8 (8.1)		7 (11.5)	1 (2.7)	8 (8.2)	
*Distant metastasis*								1.00
−	69 (84.1)	12 (75)	81 (82.6)	0.46	50 (82)	31 (83.8)	81 (82.7)	
+	13 (15.9)	4 (25)	17 (17.4)		11 (18)	6 (16.2)	17 (17.3)	

^a^ Fisher's exact test value.

While ≥200 mg/m^2^ cisplatin dose was achieved in 82 of 98 patients, <200 mg/m^2^ cisplatin dose was achieved in 16 of 98 patients. While the complete response rate was found to be 82.9% in patients achieving a dose of ≥200 mg/m^2^ cisplatin, it was found to be 75% in the patients achieving a dose of <200 mg/m^2^ cisplatin and the difference between groups was not statistically significant (*p* = 0.48) (Table 4). While local-recurrence rate was found to be 19.5% in the patients achieving a dose of ≥200 mg/m^2^ cisplatin, it was found to be 43.8% in the patients achieving a dose of <200 mg/m^2^ cisplatin and the difference between groups was statistically significant (*p* = 0.03) (Table 4). In terms of regional-recurrence rate, the difference was not statistically significant between the patients achieving a dose of ≥200 mg/m^2^ cisplatin and the patients achieving a dose of <200 mg/m^2^ cisplatin (*p* = 0.61) (Table 4). In terms of distant metastasis rate, the difference was not statistically significant between the patients achieving a dose of ≥200 mg/m^2^ cisplatin and the patients achieving a dose of <200 mg/m^2^ cisplatin (*p* = 0.46) (Table 4).

**Figure 1:**
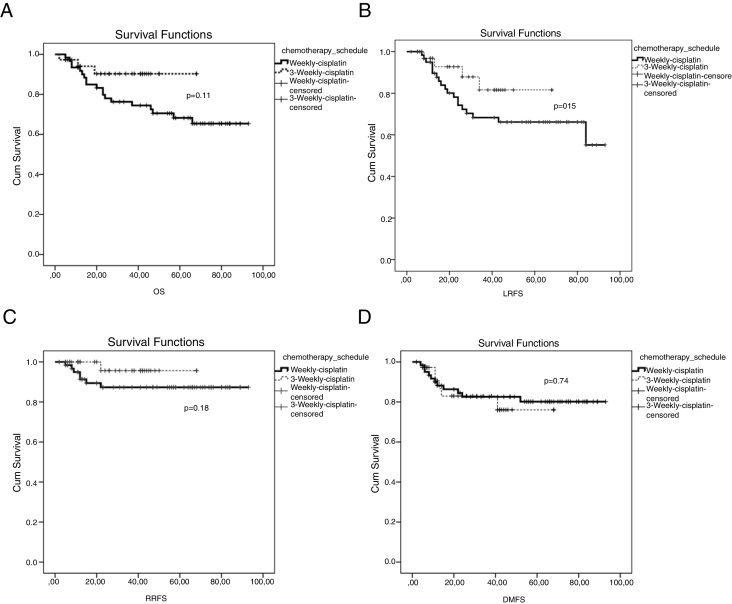
(A) Overall survival curve between 3 weekly group and weekly group; (B) locally recurrence-free survival curve between 3 weekly group and weekly group; (C) regional recurrence-free survival curve between 3 weekly group and weekly group; (D) distant metastasis-free survival curve between 3 weekly group and weekly group.

**Figure 2:**
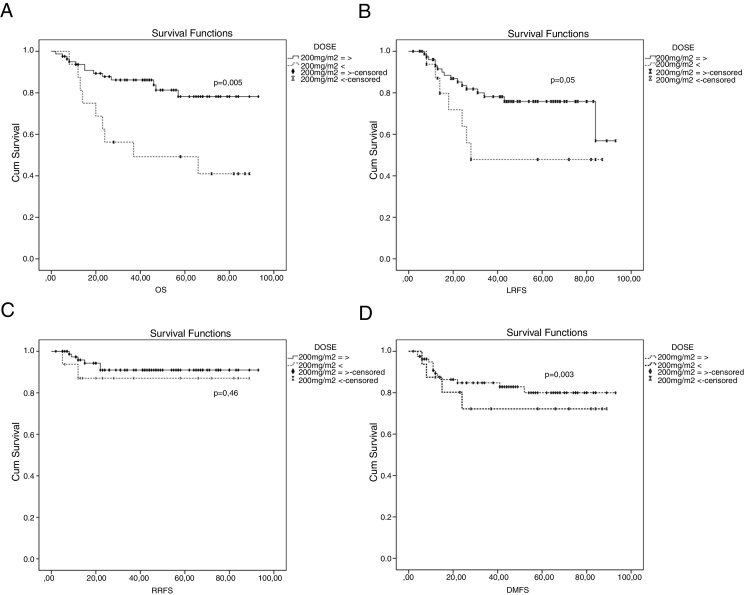
Kaplan–Meier curves for patients receiving cisplatin ≥200 mg/m^2^ and <200 mg/m^2^. (A) Overall survival curve between ≥200 mg/m^2^ and <200 mg/m^2^; (B) locally recurrence-free survival curve between ≥200 mg/m^2^ and <200 mg/m^2^; (C) regional recurrence-free survival curve between ≥200 mg/m^2^ and <200 mg/m^2^, (D) distant metastasis-free survival curve between ≥200 mg/m^2^ and <200 mg/m^2^.

The mean LRFS was found to be 70.7 months for all patients. The 5 years LRFS rates in the weekly-cisplatin group and the 3 weekly-cisplatin group were 66.2% and 81.6% respectively. There was no statistically significant difference between groups (*p* = 0.15) (Fig. 1). The 5 year LRFS rates in the patients achieving dose of ≥200 mg/m^2^ cisplatin and in the patients achieving dose of <200 mg/m^2^ cisplatin were 75.8% and 47.9% respectively. The difference between the groups was close to being statistically significant (*p* = 0.055) (Fig. 2). Clinical parameters consist of gender, age, T category, N category, TNM stage, chemotherapy schedule and cumulative cisplatin dose did not have any effect on LRFS, except for the RT technique. The LRFS was influenced by the RT technique (HR = 0.23, 95% CI: 0.068–0.787, *p* = 0.01) (Table 5).

**Table 5:** Univariate analysis for local recurrence-free survival, regional recurrence-free survival and distant metastasis-free survival.

		LRFS			RRFS			DMFS		
		HR	95% CI	*p*	HR	95% CI	*p*	HR	95% CI	*p*
Gender	Female	1.19	0.787–1.824	0.39	1.57	0.789–3.157	0.19	1.11	0.680–1.839	0.66
	Male									
Age	≤50	1.39	0.589–3.310	0.44	0.83	0.417–1.667	0.60	1.04	0.396–2.741	0.93
	>50									
T category	T 1–2	0.90	0.596–1.378	0.64	0.86	0.424–1.777	0.69	1.06	0.661–1.713	0.79
	T 3–4									
N category	N 0–1	1.24	0.814–1.905	0.31	1.00	0.501–2.005	0.99	1.06	0.661–1.713	0.79
	N 2–3									
RT technic	3D-RT	0.23	0.068–0.787	0.01	21.6	0.0–5.223	0.68	0.35	0.80–1.540	0.16
	IMRT									
TNM stage	II	1.00	0.552–1.837	0.92	1.63	0.601–4.428	0.61	1.53	0.765–3.095	0.45
	III									
	IVA									
Chemotherapy	Weekly	0.46	0.156–1.366	1.62	0.26	0.033–2.171	0.21	1.17	0.430–32.33	0.74
	3 weekly									
Cumulative dose	≥200 mg/m^2^	0.42	0.175–1.050	0.06	0.55	0.111–2.746	0.46	0.66	0.217–2.044	0.47
	<200 mg/m^2^									

3D-RT, three-dimensional radiotherapy; IMRT, intensity modulated radiotherapy; TNM, T and N categories are according to 8th edition American Joint Commission on Cancer staging system.

The mean RRFS was found to be 85.2 months for all patients. The 5 year RRFS rates in the weekly-cisplatin group and the 3 weekly cisplatin group were 87.3% and 95.7% respectively. There was no statistically significant difference between the groups (*p* = 0.18) (Fig. 1). The 5 year RRFS rates in patients achieving a dose of ≥200 mg/m^2^ cisplatin and in patients achieving a dose of <200 mg/m^2^ cisplatin were 91.0% and 87.1% respectively. There was no statistically significant difference between the groups (*p* = 0.46) (Fig. 2). The RRFS was not influenced by any clinical parameters in the univariate analysis (Table 5).

The mean DMFS was found to be 77.1 months for all patients. The 5 year RRFS rates in the weekly cisplatin group and the 3 weekly cisplatin group were 80.1% and 76.1% respectively. There was no statistically significant difference between the groups (*p* = 0.74) (Fig. 1). The 5 year DMFS rates in patients achieving a dose of ≥200 mg/m^2^ cisplatin and in patients achieving a dose of <200 mg/m^2^ cisplatin were 80% and 72.2% respectively. There was no statistically significant difference between the groups (*p* = 0.47) (Fig. 2). The DMFS was not influenced by any clinical parameters in the univariate analysis (Table 5).

The mean OS was found to be 73.6 months for all patients. The 5 year OS rates in the weekly cisplatin group and the 3 weekly cisplatin group were 68.3% and 90.3% respectively. There was no statistically significant difference between the groups (*p* = 0.11) (Fig. 1). The 5 year OS rates in patients achieving a dose of ≥200 mg/m^2^ cisplatin and in patients achieving a dose of <200 mg/m^2^ cisplatin were 78.2% and 49.2% respectively. The difference between the groups was statistically significant (*p* = 0.003) (Fig. 2).

**Figure 3:**
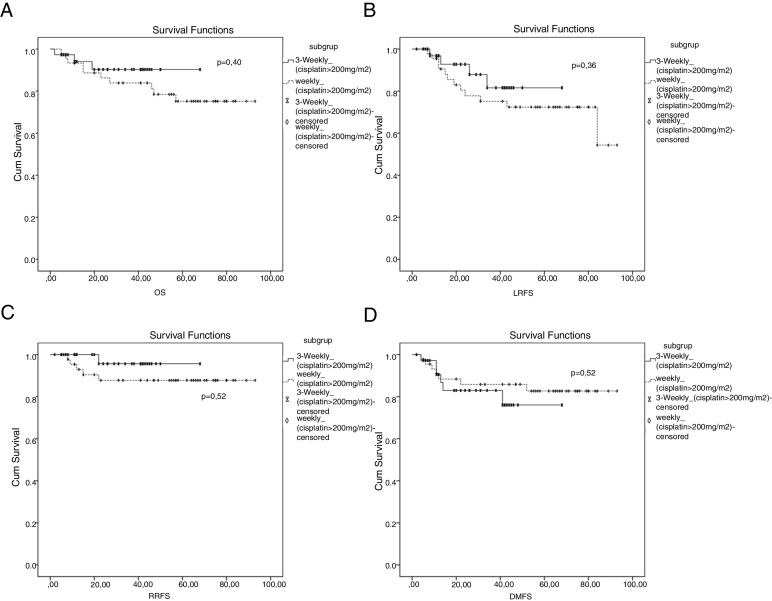
Survival curves between 3 week and weekly chemotherapy regimens in patients with cumulative cisplatin dose ≥200 mg/m^2^. (A) Overall survival curve; (B) locally recurrence-free survival; (C) regional recurrence-free survival curve; (D) distant metastasis-free survival curve.

When evaluated in subgroup analysis, there were no statistically significant differences in terms of OS, LRFS, RRFS and DMFS (*p* = 0.40, *p* = 0.36, *p* = 0.22 and *p* = 0.22, respectively) (Fig. 3) between 3-weekly and weekly chemotherapy regimens in patients with cumulative cisplatin dose of ≥200 mg/m^2^.

The cumulative dose of cisplatin and RT technique were effective on OS in univariate analysis. 2/3D conformal RT and <200 mg/m^2^ cisplatin were associated with shorter OS in univariate analysis (HR = 0.21, 95% CI: 0.071–0.628, *p* = 0.005 and HR = 0.29, 95% CI: 0.125–0.686, *p* = 0.003, respectively) (Table 6). Among the clinical parameters, only <200 mg/m^2^ cisplatin was a prognostic factor for the OS in multivariate analysis (HR = 0.36, 95% CI: 0.146–0.912, *p* = 0.03) (Table 6).

**Table 6:** Univariate and multivariate cox regression analysis for overall survival.

			Univariate			Multivariate		
		Event	HR	95% CI	*p*	HR	95% CI	*p*
Gender	Female	6	0.92	0.575–1.471	0.72			
	Male	16						
Age	<50	7	2.41	0.890–6.554	0.08			
	≥50	15						
T category	T 1–2	12	1.25	0.826–1.916	0.28			
	T 3–4	20						
N category	N 0–1	9	0.85	0.558–1.307	0.46			
	N 2–3	13						
RT technic	3D-R	4	0.21	0.071–0.628	0.005	0.33	0.105–1.087	0.06
	IMRT	18						
TNM stage	II	3	1.20	0.670–2.278	0.69			
	III	9						
	IVA	10						
Chemotherapy	Weekly	19	0.38	0.113–1.325	0.13			
	3 weekly	3						
Cumulative dose	≥200 mg/m^2^	9	0.29	0.125–0.686	0.003	0.36	0.146–0.912	0.03
	<200 mg/m^2^	13						

3D-RT, three-dimensional radiotherapy; IMRT, intensity modulated radiotherapy; TNM, T and N categories are according to 8th edition American Joint Commission on Cancer staging system.

## Discussion

In this study, all the patients had the undifferentiated, non-keratinized pathological type of nasopharyngeal carcinoma which is highly responsive to treatment. Both of these weekly and 3-weekly cisplatin groups were comparable in terms of gender, T category, N category, and RT technique.

In our center, we observed that the weekly cisplatin regimen tends to be more frequently applied in the elderly patients and in patients with comorbid disease. Weekly regimens may have been preferred by physicians to prevent the adverse effects of cisplatin in patients with lower clinical performance, comorbid disease and older patients. Various doses of cisplatin, such as 20–40 mg/m^2^, have been reported in the literature.^10^, ^23^, ^24^, ^25^, ^26^ The study of prospective NPC-9901 and NPC-9902 has shown that at least 2 cycles of 3 weekly regimens provide improvement in local recurrence-free survival and OS of cisplatin administration compared to single cycle administration.^27^ In addition, it was reported that more than 5 cisplatin applications were an independent prognostic factor in concurrent chemo-radiotherapy (2D or 3D conformal radiotherapy) applications with weekly cisplatin (40 mg/m^2^).^28^ In this study presented, we administered a cisplatin dose of 50 mg for patients receiving weekly chemotherapy. Sixteen of 61 patients who received weekly cisplatin had less than 5 cycles of chemotherapy. A significantly high (26%) local recurrence was detected in the weekly cisplatin group, which may be due to a low cumulative cisplatin dose.

In our study, 37 patients in 3-weekly cisplatin group and 45 patients in weekly cisplatin group achieved ≥200 mg/m^2^ cumulative cisplatin doses (82 of 98 patients). Cumulative dose of cisplatin is associated with long-term survival outcomes in the NPC patients treated with chemo-radiotherapy.^14^, ^29^, ^30^ NPC-9901 and NPC-9902 trials declared that at least two cycles of cisplatin (100 mg/m^2^ in every cycle) improved LRFS and OS compared to one cycle cisplatin.^14^ In the study by Loong et al.,^28^ they proved that the patients who received ≥200 mg/m^2^ cisplatin had longer survival than patients who received <200 mg/m^2^ cisplatin. In addition, Ou et al. reported that a total cisplatin dose of >300 mg/m^2^ was an independent prognostic factor for OS, DFS and DMFS in NPC patients.^31^ On the contrary, the study by Peng et al.,^32^ >240 mg/m^2^ cumulative cisplatin dose was not an independent prognostic factor for OS. Cut off value of cumulative cisplatin dose is still controversial. Our trial has also shown that the patients with >200 mg/m^2^ cisplatin dose had statistically significant long-term survival and this is compatible with the literature. In addition, LRFS in patients with ≥200 mg/m^2^ cumulative cisplatin dose were close to being statistically significant.

In this retrospective study, we observed that either weekly or 3 weekly cisplatin based concomitant chemo-radiotherapy had the similar treatment outcomes in terms of OS, DMFS, LRFS and RRFS in NPC patients. Several studies addressed that there was no difference between weekly and 3-weekly cisplatin schedules for OS, DFS and DMFS in NPC patients.^29^, ^30^, ^31^, ^32^, ^33^ The results of our study were compatible with the literature. RT technique was found as a prognostic factor in univariate analyzes for LRFS. While cumulative cisplatin doses and RT technique were found as prognostic factors in univariate analyzes for OS, only cumulative cisplatin doses was found as an independent prognostic factor for OS in multivariate analysis.

Toxicity profile seemed to be similar in both regimens. There were no statistically significant differences in terms of adverse effects consisting of hematological toxicity, mucositis, dermatitis, nausea and vomiting except for malnutrition. Mucositis was the most common adverse effect as expected. Grade 3–4 malnutrition was experienced in 33 of 37 patients in 3 weekly cisplatin group. In the study of Tao et al., Grade 3–4 mucositis were experienced more in weekly cisplatin group than 3 weekly cisplatin group.^26^ However, in our trial, there was no statistically significant difference for mucositis between the groups. In addition, none of the patients needed enteral nutrition tubes.

Limitations of the study may be the retrospective design, the expression of single center experience, an insufficient number of patients and short follow-up period.

## Conclusion

The weekly or 3-weekly cisplatin schedules in concomitant chemo-radiotherapy regimens may be preferred according to the experience in the treatment center for NPC patients. The weekly cisplatin regimen has similar treatment outcomes and adverse effects during treatment compared to the 3 weekly regimen. If patients achieve ≥200 mg/m^2^ dose of cisplatin, the weekly chemotherapy schedules may be used safely and effectively. According to the outcomes of our study, ≥200 mg/m^2^ cisplatin dose is an independent prognostic factor in NPC patients in terms of overall survival. Further prospective studies are needed to confirm the results of the present study.

## Conflicts of interest

The authors declare no conflicts of interest.
